# Spatiotemporal dynamics of OCT4 protein localization during preimplantation development in mice

**DOI:** 10.1530/REP-16-0277

**Published:** 2016-11-01

**Authors:** Atsushi Fukuda, Atsushi Mitani, Toshiyuki Miyashita, Hisato Kobayashi, Akihiro Umezawa, Hidenori Akutsu

**Affiliations:** 1 Center for Regenerative MedicineNational Research Institute for Child Health and Development, Setagaya, Tokyo, Japan; 2 Department of Molecular GeneticsKitasato University Graduate School of Medical Sciences, Minami, Sagamihara, Kanagawa, Japan; 3 NODAI Genome Research CenterTokyo University of Agriculture, Setagaya-ku, Tokyo, Japan; 4 Department of Stem Cell ResearchFukushima Medical University, 1 Hikarigaoka, Fukushima City, Fukushima, Japan

## Abstract

Spatiotemporal expression of transcription factors is crucial for genomic reprogramming. *Pou5f1 (Oct4)* is an essential transcription factor for reprogramming. A recent study reported that OCT4A, which is crucial for establishment and maintenance of pluripotent cells, is expressed in oocytes, but maternal OCT4A is dispensable for totipotency induction. Whereas another study reported that OCT4B, which is not related to pluripotency, is predominantly expressed instead of OCT4A during early preimplantation phases in mice. To determine the expression states of OCT4 in murine preimplantation embryos, we conducted in-depth expression and functional analyses. We found that pluripotency-related OCT4 mainly localizes to the cytoplasm in early preimplantation phases, with no major nuclear localization until the 8–16-cell stage despite high expression in both oocytes and early embryos. RNA-sequencing analysis using oocytes and early preimplantation embryos could not identify the splice variants creating alternative forms of OCT4 protein. Forced expression of OCT4 in zygotes by the injection of polyadenylated mRNA clearly showed nuclear localization of OCT4 protein around 3–5-fold greater than physiological levels and impaired developmental competency in a dose-dependent manner. Embryos with modest overexpression of OCT4 could develop to the 16-cell stage; however, more than 50% of the embryos were arrested at this stage, similar to the results for OCT4 depletion. In contrast, extensive overexpression of OCT4 resulted in complete arrest at the 2-cell stage accompanied by downregulation of zygotically activated genes and repetitive elements related to the totipotent state. These results demonstrated that OCT4 protein localization was spatiotemporally altered during preimplantation development, and strict control of Oct4 protein levels was essential for proper totipotential reprogramming.

## Introduction

Cellular reprogramming and differentiation are accompanied by dynamic transcriptional changes and transcription factors play central roles in both processes (Buganim *et al*. 2013). Dramatic alterations of cellular fates occur during preimplantation embryo development (Burton & Torres-Padilla 2014). In mice, after fertilization, most maternal factors are degraded and zygotic gene activation (ZGA) occurs by the 2-cell stage (Hamatani *et al*. 2004). An important transcription factor responsible for sustaining pluripotency is the POU5F1 protein (also known as OCT4) (Nichols *et al*. 1998). In zebrafish, Oct4 is crucial for driving ZGA (Lee *et al*. 2013, Leichsenring *et al*. 2013). In mammals, although *Oct4* expression defines pluripotent stem cell fates dose-dependently (Niwa *et al*. 2000), maternal *Oct4* was shown to be dispensable for *in vivo* totipotential reprogramming during the preimplantation phase (Wu *et al*. 2013). However, Marti and coworkers showed that *Oct4* has two isoforms and *Oct4B*, which is not associated with pluripotency, was found to be predominantly expressed, whereas *Oct4A* was not expressed during early preimplantation phases of mice (Marti *et al*. 2013).

The presence of *OCT4B* transcripts was reported in some human cell lines (Atlasi *et al*. 2008). In mice, *Oct4B* transcript was found in embryonic stem cells (Guo *et al*. 2012). The differing regions in the two Oct4 isoforms were the first exon in both mice and humans (Atlasi *et al*. 2008, Guo *et al*. 2012). However, the Oct4 variants have not been identified in preimplantation phases in mice. These studies led us to question whether pluripotency-related *Oct4* is substantially expressed in murine early preimplantation embryos. In this study, we conducted deep expression analysis and dose-dependent functional analyses of Oct4 in the preimplantation stages in mice.

## Materials and methods

### Oocyte collection and embryo manipulation

All mice were maintained and used in accordance with the Guidelines for the Care and Use of Laboratory Animals of the Japanese Association for Laboratory Animal Science and the National Research Institute for Child Health and Development of Japan (A2006-009-bib9). Adult female (8–12 weeks of age) and male (8–16 weeks) B6D2F1 mice were purchased from CLEA Japan (Tokyo, Japan), and oocytes were collected following standard methods. All embryos were cultured in KSOM (Millipore, Billerica, MA, USA) medium at 37°C and 5% CO_2_.

All microinjection experiments were carried out based on previous reports (Fukuda *et al*. 2014). In brief, *in vitro* fertilized embryos at 1–1.5 h after sperm input were washed in M2 medium and incubated for 1 h. mRNA or siRNA injection was conducted using a PiezoDrive (Prime Tech, Ibaraki, Japan) and the embryos were cultured in KSOM medium.

### *In vitro* mRNA synthesis and siRNA preparation

The coding regions of *Oct4* mRNA in 4-cell embryos were amplified by PCR amplification using KOD-Plus-Neo DNA polymerase (Toyobo, Osaka, Japan) with T7-containing forward and reverse primers with poly T (Supplementary Table 1, see section on supplementary data given at the end of this article). Using the DNA templates, *Oct4* mRNA was generated by *in vitro* transcription using an mMessage kit (Life Technologies) following manufacturer’s instructions. *Oct4* mRNA concentrations were adjusted to 50, 100 and 200 ng/µL. The same number of mRNA molecules was found in 200 ng/µL *Oct4* mRNA and 130 ng/µL *EGFP* mRNA used for injection. siRNAs targeting *Oct4* (sense: 5ʹ-GUU CGA GUA UGG UUC UGU ATT-3ʹ, antisense: 5ʹ-UAC AGA ACC AUA CUC GAA CCA-3ʹ) and a negative control (silencer select negative control, #4390846; Ambion) were purchased from Life Technologies. Each siRNA (25 ng/mL) was injected into zygotes.

### RT-PCR analysis

Total RNA from 50 GV oocytes, 50 MII oocytes, 50 1-cell, 100 2-cell, 130 4-cell, 40 morulae and 30 blastocysts was extracted using an RNeasy Micro kit (Qiagen). cDNA was synthesized using SuperScript III and random hexamers (Life Technologies) and used for RT-PCR analysis. PCR was conducted using KOD FX neo polymerase (Toyobo) according to manufacturer’s instruction. PCR cycle number was 45 with a 60°C annealing step.

### qPCR analysis of single and pooled embryos

Each single embryo was lysed and reverse transcription was conducted using the Single Cell to CT kit (Life Technologies) according to manufacturer’s instruction. Total RNA of pooled embryos was extracted using an RNeasy Micro kit (Qiagen) and cDNA was synthesized using SuperScript III and random hexamers (Life Technologies) according to manufacturer’s instruction. The synthesized cDNA was used for TaqMan gene expression analysis (Life Technologies) in all qPCR assays except for major satellite and MERVL quantification, which were carried out using a SYBR Green assay (Bio-Rad). For MERVL normalization, β-actin was used for the internal control. For strand-specific reverse transcription of major satellites, 2 μM of each primer (forward transcript detection: CAT ATT CCA GGT CCT TCA GTG TGC; reverse transcript detection: GAC GAC TTG AAA AAT GAC GAA ATC) was used instead of random hexamers. The same number of cells was used for the assay to be normalized to cell number. The TaqMan probes used in this study are shown in Supplementary Table 1. The data of Supplementary Fig. 7 were analyzed using the R function ‘hclust’ (https://www.r-project.org/) to produce unsupervised clustering.

### Immunofluorescence (IF)

Antibodies for Oct4-C10 (1:500; Santa Cruz Biotechnology), Oct4-N20 (1:500; Santa Cruz), H3K9me3 (ab8898; Abcam), RING1B (1:500; Cell Signaling Technologies), 5-methylcytosine (1:500; Eurogentec, Liége, Belgium) and 5-hydroxycytosine (1:500; Active Motif, Tokyo, Japan) were used for IF.

To prepare the samples, the zona pellucida was removed by acid Tyrode’s solution (Millipore) and washed in PBS containing 0.1% polyvinyl alcohol (PBS-PVA) before fixation. For Oct4, the embryos were fixed in 2% PFA for 10 min followed by permeabilization in 0.25% Triton-X for 10 min at room temperature. For H3K9me3 and RING1B, fixation and permeabilization were conducted simultaneously for 5 min at room temperature. For 5-methylcytosine and 5-hydroxycytosine staining, the samples were fixed followed by permeabilization as described above and were subjected to 2.4 N HCl treatment (in PBS-0.5% PVA) for 20 min at room temperature and then neutralized in 100 mM Tris–HCl in PBS-PVA for 15 min.

After fixation and permeabilization, the samples were blocked in PBS containing 1% BSA (blocking buffer) for 1 h and incubated with antibodies overnight at 4°C. Embryos were washed with PBS containing PBS-PVA and then incubated for 1 h at room temperature with Alexa Flour 546-, 633-conjugated IgG secondary antibodies, or 546-conjugated IgG2b (for C-10 antibody) (Life Technologies, 1:500). After the embryos were washed with PBS-PVA and attached onto cover slides, the nuclei were stained with DAPI.

### Quantification of nuclear OCT4 protein

To quantify OCT4 nuclear protein levels by IF, the same laser intensity was applied to all samples. Three-dimensional images were constructed from *Z*-sections in the LSM Image Browser (Carl Zeiss). The total signal intensities of the maximum projection and nuclear area determined by DAPI-positive regions were calculated using ImageJ software (http://imagej.nih.gov/ij/). Statistical analysis was performed using Student’s *t*-test.

### Western blotting (WB)

Oocytes and embryos were washed in PBS-PVA and lysed (in sample buffer containing SDS and 2-Me) and heated for 5 min at 95°C. The lysates were subjected to SDS-PAGE using e-PAGEL (ATTO, Tokyo, Japan). For native-PAGE, the samples were lysed in native sample buffer (Bio-Rad) and vortexed. The lysates were then subjected to PAGE using e-PAGEL (ATTO) on ice. The transferred membranes were washed in TBS containing 0.1% Tween 20 (TBS-T) and blocked in 5% skim milk (Morinaga, Tokyo, Japan) in TBS-T for 1 h. The membranes were incubated with OCT4-C10 antibody (1:200, Santa Cruz) overnight at 4°C, washed and incubated with a mouse HRP-conjugated secondary antibody (1:2000, Sigma-Aldrich) for 1 h. Immunoblots were visualized using SuperSignal chemiluminescent substrate (Thermo Scientific) and an ImageQuant LAS4000 system (GE Healthcare). After image capture, the membranes were washed, blocked and incubated with an anti-TUBULIN (1:2000, Sigma-Aldrich), GAPDH (1:2000, Wako) or RNAPII (1:2000, Active Motif) antibody. The membranes were then washed and visualized using the same method.

For the collection of 100 morula cells, the zona pellucida of each embryo was removed and the embryos were incubated in PBS containing polyvinyl alcohol (0.1%) and cytochalasin B (5 µg/mL) at room temperature for 10 min. Morula cells were collected using a micromanipulator.

For examination of phosphorylation state, we conducted Phos-tag SDS-PAGE. The samples were prepared according to the above method. In the PAGE procedures, the gel (SuperSep Phos-tag (50 μmol/L), 12.5%) was used according to manufacturer’s instruction.

### Separation of nuclei and cytoplasm

The embryos at 1- and 2-cell stages were incubated in M2 medium containing cytochalasin B (10 μg/mL) and nocodazole (1 μg/mL) for 10 min at 37°C. Enucleation was performed using a Piezo Micro Manipulation system in the above medium. The collected nuclei and cytoplasm were subjected to WB analysis.

### BrdU incorporation assay

The embryos were incubated with 10 μM BrdU (Sigma) from 23 to 33 h after insemination. The samples were subjected to the same procedure as IF using 5-methylacytosine/hydroxycytosine antibodies. Anti-BrdU (1:100, Abcam) and anti-mouse 546 IgG secondary antibody were used and the images were captured by LSM510 laser scanning confocal microscopy (Carl Zeiss). The signal intensity was quantified using Image J software.

### DNA- and RNA-FISH

The zona pellucida was removed and the samples were washed in PBS-PVA. The samples were fixed and permeabilized simultaneously (in 2% PFA and 0.25% Triton-X in PBS-PVA) for 5 min at room temperature and placed on a coverslip. For RNA-FISH, hybridization buffer containing a locked nucleic acid (LNA) probe (Exiqon, Vedbaek, Denmark) targeting major satellites was applied to the slide as reported previously (Probst *et al*. 2010), and incubated overnight at 37°C in a humidified atmosphere. After washing twice with 2× SSC containing 50% formamide and 2× SSC with 0.05% Tween 20, the embryos were stained with DAPI.

For DNA-FISH, the samples after fixation and permeabilization were subjected to RNaseA treatment for 1 h at 37°C. After washing with PBS, the samples were incubated with 0.1 N HCl in 0.1% Triton-X-PBS for 10 min on ice. The samples were washed and hybridized with the LNA probe at 85°C for 10 min and incubated overnight at 37°C. The coverslips were washed twice with 2× SSC containing 50% formamide and 2× SSC and stained with DAPI. Fluorescence was visualized using the LSM510.

### Transcriptome analysis

Total RNA following DNaseI treatment was extracted using an RNeasy Micro kit (Qiagen). For construction of sequencing libraries, we used an Ovation Single Cell RNA-Seq System (NuGEN, West Cumbria, UK). Strand-specific, paired-end sequencing (length: 100 bp) was performed using a HiSeq system (Illumina, Inc; San Diego, CA, USA), with six samples per lane. BAM format data yielded by Tophat 2.0.11 (bowtie2-2.2.1) were subjected to successive analyses using Cufflinks-2.2.1. The counts of raw reads allocated for each gene/transcript that linked to UCSC transcripts were normalized to the fragments per kilobase value per million mapped reads (FPKM) value (Cufflinks-2.2.1). Normalized values were described as log2 values. The genes with >5 FPKM in either Oct4-OE or control (Egfp-OE) groups were filtered and used for differentially expressed genes screening. For Fig. 2C, of 6851 genes showing Oct4 binding (Chen *et al*. 2008), 2512 were filtered (>5 FPKM in either group) and plotted. The raw data were deposited in Gene Expression Omnibus (accession I.D. of SRA: SRR3103036, SRR3103038, SRR310340 and SRR310341.).

### Gene set enrichment analysis

The genes expressed at 1- and 2-cell stages were downloaded from http://dbtmee.hgc.jp (Park *et al*. 2013). The genes with >3-fold upregulation in 2-cell embryos were used as ZGA-associated genes (2720). The ZGA-related genes were used for comparison with the genes filtered in the transcriptome analysis using Gene set enrichment analysis (http://www.broadinstitute.org/gsea/index.jsp).

### Variant calling for the Oct4 gene from RNA-seq data

The TopHat2 reads aligned to the mouse reference genome were used for variant calling. Genomic regions of the exons of the *Oct4* (*Pou5f1*) gene were downloaded from the UCSC table browser (http://genome.ucsc.edu/). Multiple identical reads from the exact same fragment on *Oct4* were marked as duplicates by Picard Tools version 1.119 (http://picard.sourceforge.net). Variant calling with the Unified Genotyper was performed by the Genome Analysis Toolkit (GATK) Lite version 2.3-9 from the Broad Institute (https://www.broadinstitute.org/gatk/). The called variants were not subjected to a filtering step to avoid filtering low-quality true-positive variants. The effects of the variants on *Oct4* were predicted using SnpEff version 3.6 (Flicek *et al*. 2014), based on the database Ensembl GRCm38.75 (Cingolani *et al*. 2012).

### Differential transcription analysis in repetitive sequences

Reads were aligned to the mouse reference genome mm10 allowing up to 20 multiple mappings in the genome using TopHat2. Genomic regions of repeat elements were obtained with the RepeatMasker track in the UCSC table browser (http://genome.ucsc.edu/). Expression values of repeat elements were calculated as FPKM using Cuffdiff of the Cufflinks suite.

## Results

### Oct4 mRNA and protein expression states in preimplantation embryos

**Figure 1:**
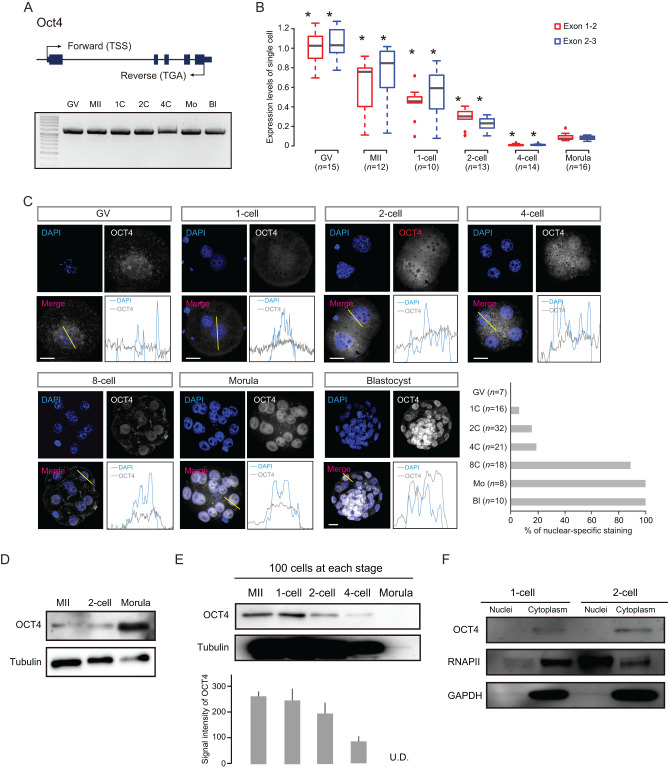
In-depth expression analysis of Oct4 mRNA and protein in oocytes and preimplantation embryos. (A) RT-PCR analysis from the transcription start site (TSS) to translation termination site in oocytes and preimplantation embryos. Two independent experiments were conducted. (B) Single-cell qPCR analysis for two *Oct4* exon junction regions. n, the number of cells used for the analysis. *P < 3.0 E-7 by Student’s *t*-test using average expression levels (compared with morula). (C) IF analysis using C-10 antibody. Representative images with intensity plots and the ratios of nuclear localization are shown. Scale bars = 20 μm. (D) SDS-WB analysis using whole cell lysates. Three independent experiments using over 30 embryos were conducted. (E) Absolute quantification of OCT4 protein by SDS-WB analysis. One hundred cells were prepared in each stage. TUBLIN was used as a loading control. The bar graph shows average OCT4 signal intensity in three independent experiments and error bars show standard errors (SE). U.D. (undetectable). (F) SDS-WB analysis using cytoplasm and nuclei lysates from early preimplantation embryos. Two independent experiments were conducted. GAPDH and RNAPII were used as loading controls.

We first examined whether full-length *Oct4A* transcripts were present from oocyte to preimplantation phases. Reverse transcription polymerase chain reaction (RT-PCR) analysis demonstrated that the protein-coding region (CDS: Bbib68268.1) was expressed during these phases (Fig. 1A). Quantitative PCR (q-PCR) analysis using single cells from oocyte to morula stages revealed that *Oct4* levels were highest in the oocyte and gradually decreased after fertilization, reaching minimum levels at the 4-cell stage (Fig. 1B and Supplementary Fig. 1). Although expression in morula cells was slightly upregulated compared with that of 4-cell stage cells, it was significantly lower than in oocytes (Fig. 1B and Supplementary Fig. 1). Thus, although *Oct4* expression levels differed in each stage, the coding region of the mRNA was expressed from oocyte to late preimplantation phases.

To generate OCT4 protein profiles from oocyte to blastocyst stages, we used immunofluorescence (IF) with an OCT4A-specific antibody (referred to as C10) that was prevalently used for OCT4 protein detection in mice and humans (Wu *et al*. 2013). We first examined the expression patterns. Notably, at oocyte to 4-cell stages, the OCT4A staining patterns using the C10 antibody (Oct4-C10) did not show the nuclear localization typically seen in pluripotent stem cells (Fig. 1C). At the 8-cell stage, Oct4 nuclear localization became partially apparent (88% embryos) and all examined morulae and blastocysts showed robust Oct4-C10 expression with nuclear localization (Fig. 1C). Next, to quantify the nuclear protein levels of OCT4, the same laser intensity was applied to each sample (adjusted to the morula stage). The signals of oocytes, 1-, 2-, and 4-cell embryos decreased (Supplementary Fig. 2), indicating that the OCT4 nuclear expression was high at the late stage of preimplantation; however, the mRNA levels in the morula stage were significantly lower than those of early preimplantation cells (Fig. 1B).

**Figure 2:**
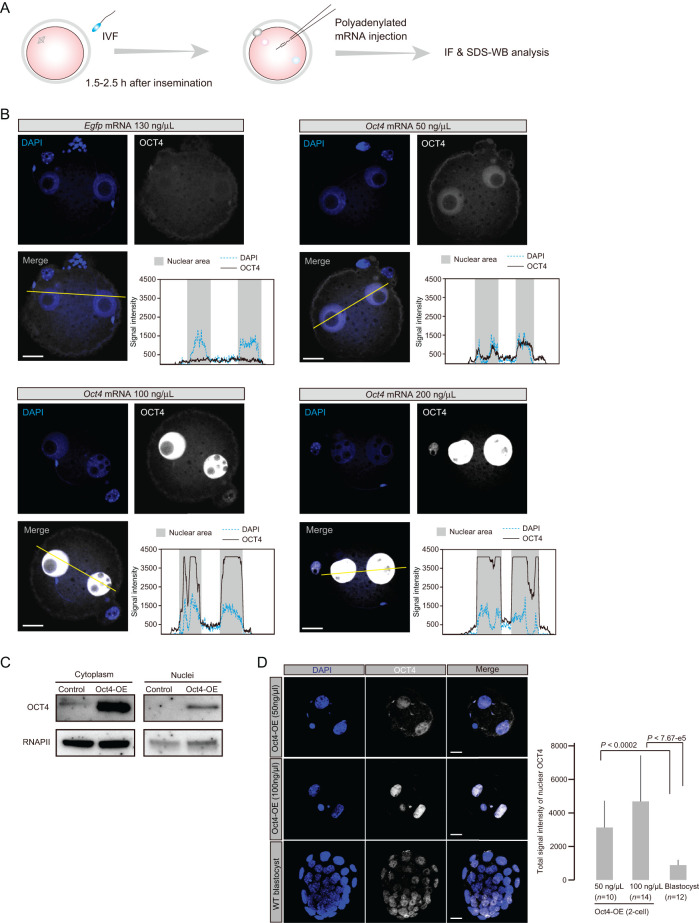
Exogenous *Oct4* expression shows robust nuclear localization. (A) Experimental scheme for the production of OCT4 overexpressing and depleted embryos. (B) IF analysis of embryos injected with *Oct4* mRNA of various concentrations at 6 h after injection. Representative images of maximum projection and intensity plots in OCT4-OE and control embryos were shown. The same strong laser intensity was applied to all samples. Dotted and solid lines indicate DAPI and OCT4 signals respectively. The scale bars represent 20 μm. (C) SDS-WB analysis using cytoplasmic and nuclear lysates from zygotes of OCT4-OE and control samples. Two independent experiments were conducted. RNAPII was used as a loading control. (D) Quantification of OCT4 nuclear protein levels. The same laser intensity was applied to all samples, and the signal intensities were measured using ImageJ. Representative images are presented, and quantified signals are shown as bar graph. n indicates the number of cells analyzed for each measurement. The *P*-values were calculated by Student’s *t*-test. Error bars indicate the standard deviation. Scale bars = 20 μm.

We also examined OCT4B expression states using a specific antibody (Oct4-N20). In contrast to Oct4-C10 results, we observed nuclear localized Oct4-N20 signals in 68% GV oocytes and 70% 1-cell embryos (Supplementary Fig. 3). However, we did not observe robust nuclear Oct4-N20 staining from 2-cells onward in most embryos (Supplementary Fig. 3 and not shown), nor was observed with another Oct4-N20 antibody with a different lot number (not shown). We sought the splice variants of *Oct4* using RNA deep sequencing (RNA-seq) data of germinal vesicle (GV) oocytes (Fukuda *et al*. 2015). However, we did not identify the clear splice variants coding for *Oct4B* identified in human and mouse pluripotent stem cells (Atlasi *et al*. 2008) (Supplementary Fig. 4). Considering the RNA-seq results, Oct4-N20 might therefore identify a nonspecific protein in mice. Although these results were inconsistent with a previous report (Marti *et al*. 2013), Marti and coworkers did not presented the number of embryos in the analysis using Oct4-N20 antibody, and we could not directly compare our results with their findings.

To confirm the protein expression states of OCT4 by a different approach, we conducted Western blotting (WB) analysis using Oct4-C10 (hereafter, we refer to OCT4A as OCT4). Notably, OCT4 protein was detected in MII oocytes and 2-cell embryos as well as morulae (Fig. 1D), rejecting the possibility that *Oct4* mRNA was not translated until the morula stage. Next, to examine absolute expression levels, we prepared 100 cells from oocyte to morula stages respectively (i.e., 100 oocytes, 100 zygotes, 50 2-cell embryos, 25 4-cell embryos and 7–10 morulae), and performed SDS-WB analysis. This showed that OCT4 protein levels from oocytes to 4-cells were markedly higher than in morulae (Fig. 1E), indicating that the protein level per cell was not dramatically increased in morulae exhibiting nuclear localization of the protein by IF.

The possibility for the inconsistent results of IF and SDS-WB might be different protein localization. Generally, Oct4 is localized in the nuclei in pluripotent cells (Marti *et al*. 2013, Wu *et al*. 2013, Yasuhara *et al*. 2013). We examined whether the protein detected by SDS-WB analysis in early preimplantation embryos was derived from the nucleus or cytoplasm. The nuclei of 1- and 2-cell embryos were removed to separate the nuclei and cytoplasm (Supplementary Fig. 5). SDS-WB analysis revealed that the majority of the Oct4 protein was localized in the cytoplasm in both stages (Fig. 1F). Taken together, we concluded that in oocytes and early preimplantation embryos, OCT4 protein mainly localized to the cytoplasm but localized specifically to the nucleus at the late preimplantation stage.

### Exogenous OCT4 expression in zygotes shows nuclear localization

To gain further insight into OCT4 protein expression states, we constructed Oct4 overexpressing embryos (Oct4-OE) by polyadenylated mRNA injection of various concentrations (50, 100 and 200 ng/μL). For an injection control, to allow for the toxicity of injected mRNA, we prepared 130 ng/μL *Egfp* mRNA that corresponded to 200 ng/μL *Oct4* mRNA molecules. Injection was performed 1.5–2.5 h after insemination to adjust fertilization and injection timing (Fig. 2A).

We first examined whether exogenous OCT4 protein could localize to nuclei at the zygote stage by IF analysis. Zygote nuclei exhibited dose-dependent IF signal intensity (Fig. 2B). Interestingly, we did not observe marked cytoplasmic expression even when high-intensity laser excitation was applied to Oct4-OE (Fig. 2B). However, SDS-WB analysis using Oct4-OE (100 ng/μL) zygotes revealed high expression of exogenous Oct4 protein in both the nucleus and cytoplasm (Fig. 2C), indicating that the C-10 antibody could not identify cytoplasmic OCT4 protein in the IF assay.

Next, we quantified the total levels of OCT4 nuclear protein. We first applied SDS-WB using the nuclear fraction of Oct4-OE at the 2-cell stage and ES cells with RNAPII as the nuclear internal control. However, we found that RNAPII expression states differed between 2-cell embryos and ES cells: RNAPII of ES cells was robustly detected in both the nuclear and cytoplasmic fractions (not shown), consistent with a recent report using DT40 cells (Hsin *et al*. 2014). Therefore, we performed quantitative analysis using IF data with the C-10 antibody and compared the observed levels of Oc4-OE with those of blastocysts, which are pluripotent cells. Comparison with blastocyst nuclei showed that the total nuclear levels of OCT4 protein in Oct4-OE (50 ng/μL) and (100 ng/μL) were more than 3.5- and 5.3-fold increased respectively (Fig. 2D). These results revealed that the levels of exogenously expressed OCT4 nuclear protein were higher than those under physiological conditions.

Taken together, these results indicate that OCT4 protein localization was spatiotemporally altered between the early and late preimplantation phases. Given that exogenous OCT4 protein could be present in the nucleus, the conformation of OCT4 protein under physiological conditions at early preimplantation phases might differ from that in late preimplantation embryos or pluripotent cells.

### Exogenous Oct4 expression impairs developmental competency in a dose-dependent manner

**Figure 3:**
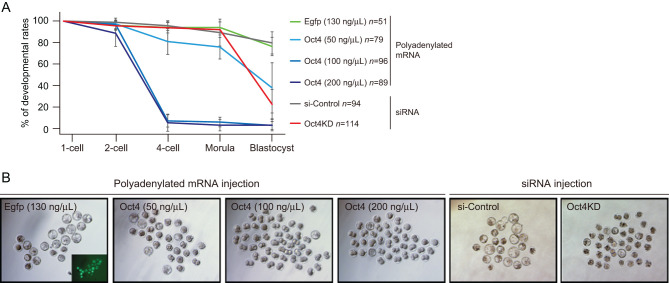
Developmental ability of *Oct4* overexpressing and depleted embryos. (A and B) Developmental ability of Oct4-OEs and Oct4KD embryos (A). Representative images at day 4 after fertilization (B).

Because *Oct4* regulates cell fates in a dose-dependent manner in mammals (Niwa *et al*. 2000), we wondered whether the lack of nuclear OCT4 protein in early preimplantation phases had biological meaning during reprogramming into totipotency. To gain insight into the effect of exogenous expression of Oct4 on development, we cultured Oct4-OE. Within 24 h after insemination, over 95% Oct4-OE and controls had developed to the 2-cell stage (Fig. 3A). The developmental rate to the blastocyst stage in the control group was 77% (Fig. 3A and B). However, at 48 h, >95% Oct4-OE high concentration groups (100 and 200 ng/μL) were arrested at the 2-cell stage (Fig. 3A and B). In addition, although over 80% low Oct4-OE group developed to the 4-cell stage, only 38% embryos developed to blastocyst stages (Fig. 3A and B). In contrast, Oct4 depletion by siRNA injection (Oct4KD) also caused developmental failure of >60% of embryos after the morula stage, consistent with a previous study (Tan *et al*. 2013) (Fig. 3A, B, and Supplementary Fig. 6). Taken together, these results indicated that Oct4 overexpression with nuclear localization from the zygote stage negatively influenced developmental competency in a dose-dependent manner.

### Effect of transcriptome state on Oct4-overexpressing embryos

To determine the cause of developmental failures by Oct4-OE (50 ng/μL: low), we conducted gene expression analysis. We first examined the gene expression states in the morula cells in Oct4-OE (low) and Oct4KD embryos. Because most of the transcription factors related with pluripotency commence transcription at the morula stage (Guo *et al*. 2010), we carried out qPCR using 21 pluripotency- and 7 epigenetic modification- related genes. Unsupervised clustering analysis showed that the Oct4-OE (low) group was categorized with the control groups (si-RNA control and Egfp-OE), whereas Oct4KD was clearly divided (Supplementary Fig. 7A). The lack of total *Oct4* mRNA and protein upregulation in Oct4-OE (low) suggested that external *Oct4* was degraded by the morula stage (Supplementary Fig. 7B and C). The developmental failure of Oct4-OE (low) was not obviously caused by dysregulation of major transcription factors associated with pluripotency, whereas *Cdx2*, *Tet2* and *Uhrf1* were markedly repressed and *Eomes* was upregulated in Oct4KD embryos (Supplementary Fig. 7B). Thus, although the transcriptional states differed, both treatments led to similar embryo phenotypes (Fig. 3A and B), demonstrating that strict expression levels of *Oct4* are required for proper development *in vivo*.

**Figure 4:**
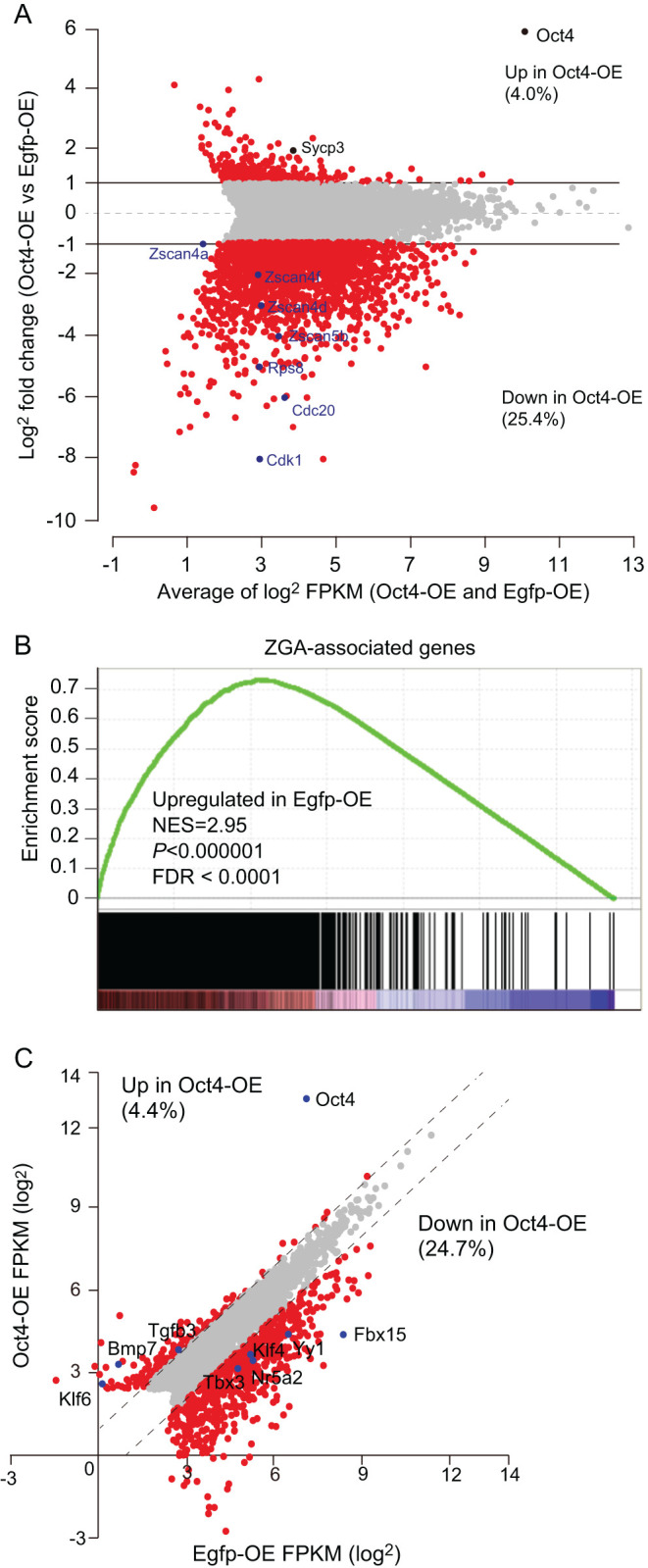
Transcriptome analysis by RNA-seq in Oct4-OE. (A) MA plot from RNA-seq data displaying differentially expressed genes (DEGs) in Oct4-OE and Egfp-OE at 30 h after insemination. The genes with >5 FPKM (6763) in either groups were used for analysis. Red circles show DEGs with over two-fold change. (B) Gene set enrichment analysis against ZGA-associated genes. The genes used for the assay was selected as described above. The ZGA genes that were expressed at 2-cell but not 1-cell stages were selected using published data (Supplementary Methods). (C) Expression states of Oct4 binding genes. Previously reported lists of Oct4 binding genes in ES cells were used (see methods). We plotted 2512 genes with >5 FPKM in either groups. Red circles show DEGs with over two-fold change.

Our study showed that high levels of exogenous OCT4 expression caused complete 2-cell arrest, implying that ectopic expression of OCT4 might affect ZGA. To elucidate the cause of the 2-cell arrest in Oct4-OE, we examined transcriptional states at the 2-cell stages of Oct4-OE (100 ng/μL: high) and controls using RNA-seq at 30 h after insemination. In Oct4-OE, 29.4% genes with >5 FPKM were differentially expressed over two-fold. There were markedly more downregulated than upregulated genes in Oct4-OE (1718 (25.4%) vs 268 (4.0%) genes respectively; Fig. 4A). Although the differentially expressed genes (DEGs) were randomly distributed among all chromosomes (Supplementary Fig. 8), ribosome-related genes and *Zscan4* family genes (Zeng & Schultz 2005, Zalzman *et al*. 2010), which were specifically expressed at the 2-cell stage, were downregulated (Fig. 4A and Supplementary Fig. 9). Furthermore, gene set enrichment analysis revealed that Oct4-OE downregulated genes were significantly enriched for ZGA-related genes (Fig. 4B). Thus, ectopic expression of *Oct4* caused the disruption of a 2-cell-specific transcriptional program including ZGA.

We then asked whether the DEGs could be associated with the OCT4 binding potential. As chromatin immunoprecipitation assays for OCT4 using preimplantation embryos are technically difficult, we used known gene lists for which bindings were validated in ES cells (Chen *et al*. 2008). Out of 2512 genes, 4.4 % (111) and 24.7% (620) genes were up- and downregulated over two-fold in Oct4-OE (Fig. 4C). Notably, *Fbx15*, whose expression pattern correlates to that of *Oct4* in ES cells (Tokuzawa *et al*. 2003), was strikingly repressed. Taken together, these results suggest that ectopic *Oct4* expression induced changes in global transcription and arrested development, resulting in the downregulation of many genes.

### Silencing of totipotency-related repetitive elements by exogenous Oct4 expression

Recent studies indicate that totipotency-associated cells are rarely found among pluripotent stem cells, which exhibit an OCT4 protein negative state and the activation of endogenous retrovirus (MERVL) transcription (Macfarlan *et al*. 2012, Ishiuchi *et al*. 2015). In addition, major satellite transcripts have also been found to be specifically expressed at the 2-cell stage, and their repression led to developmental arrest at the 2-cell stage (Probst *et al*. 2010, Casanova *et al*. 2013). These studies promoted us to investigate the transcription states of repeats in Oct4-OE (high).

**Figure 5:**
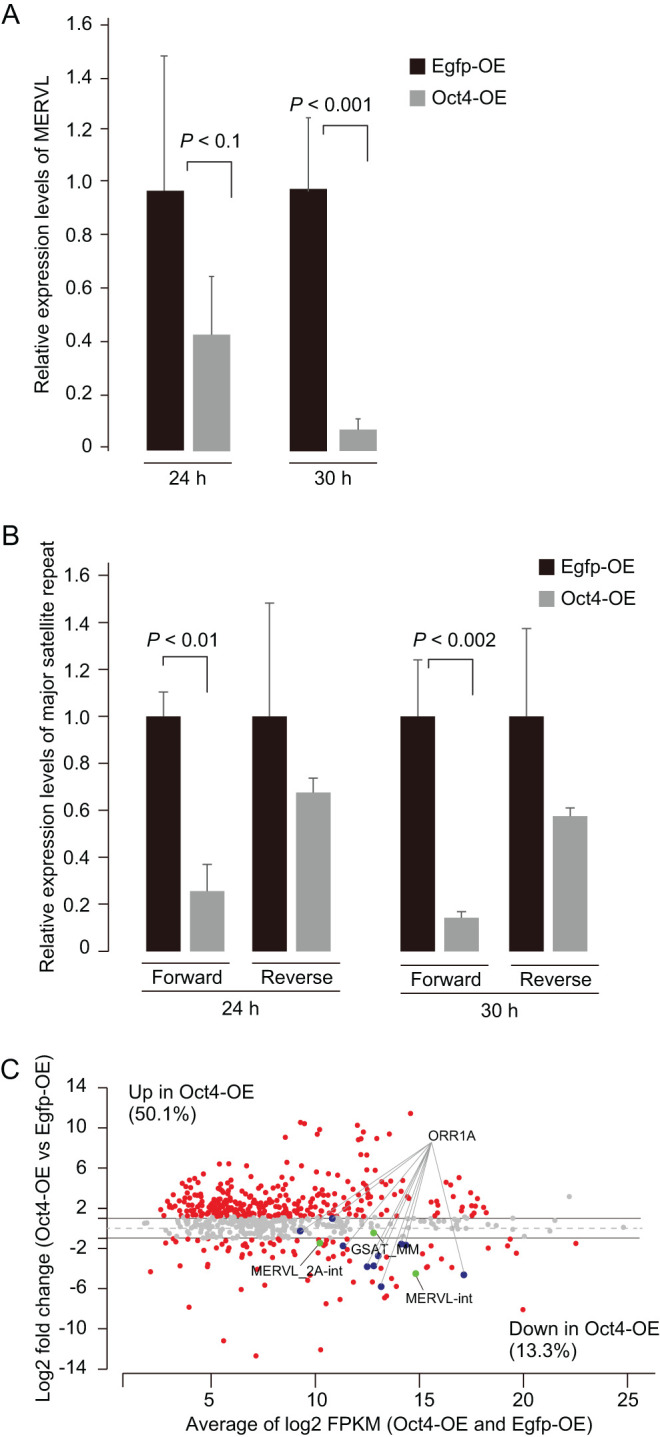
Expression states in totipotency-related repetitive transcripts. (A) qPCR analysis of MERVL transcripts in Oct4-OEs and Egfp-OE at 24 and 30 h after insemination. The target gene was normalized by b-actin. (B) Strand-specific qPCR for major satellite transcripts in Oct4-OEs and Egfp-OE at 24 and 30 h after insemination. The ten 2-cell embryos (20 cells) were used for each qPCR assay. Three biological replicates were used. P-values were based on *t*-tests. Error bars show standard deviations (A and B). (C) MA plot from RNA-seq showing differentially expressed repetitive transcripts. A total of 655 repeats showed >5 FPKM.

The qPCR analysis revealed that MERVL expression levels in Oct4-OE were significantly reduced to <10% of control at 30 h after insemination (Fig. 5A). For the major satellite transcripts, repression was greater among forward transcripts than reverse (forward: <30% of control vs reverse: <68%, Fig. 5B), and we confirmed these results using strand-specific RNA-FISH (Supplementary Fig. 10).

Notably, comprehensive analysis of repetitive elements by RNA-seq revealed that 50% of repetitive sequence types were upregulated >2-fold in Oct4-OE (high) (Fig. 5C), whereas major satellites and MERVL were downregulated (Fig. 5C). We also found that ORR1a retroviral elements, which were upregulated during preimplantation development (Peaston *et al*. 2004), were repressed in Oct4-OE as well (Fig. 5C). Taken together, OCT4 overexpression altered not only the proper ZGA program but also 2-cell-specific transcript expression at repetitive elements.

### High levels of exogenous Oct4 expression induce aberrant chromatin conformation

**Figure 6:**
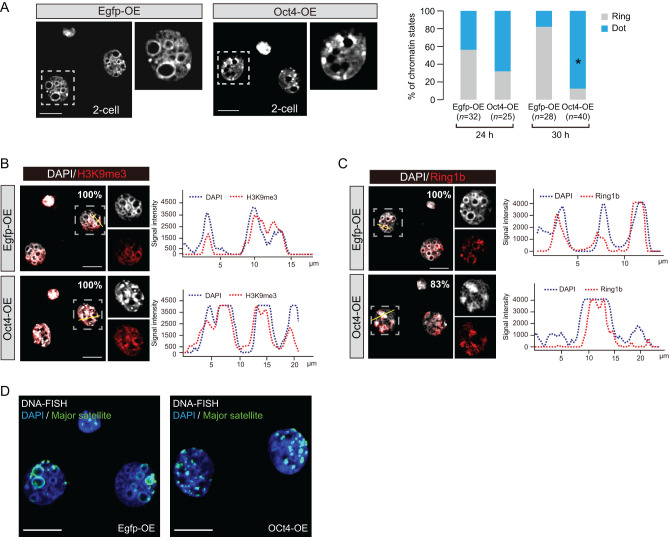
High-level nuclear OCT4 disrupts 2-cell specific chromatin conformation. (A) PHC formation states visualized by DAPI intense staining. PHC states were divided into two types based on the staining patterns. Representative images are shown. *P-values < 0.00001 by Fisher’s exact test. Scale bar represents 10 μm. ‘n’ indicates the number of nuclei examined. (B and C) IF analysis of H3K9me3 (B) and Ring1b (C) in Oct4-OEs and Egfp-OE at 30 h after insemination. H3K9me3 and Ring1b predominantly localized on PHC of maternal and paternal genomes respectively. Percentages indicate the embryos showing DAPI intense areas and H3K9me3 or Ring1b overlapping in at least one region. Over 10 embryos were analyzed. (D) DNA-FISH for major satellite regions. Embryos were analyzed at 30 h after insemination.

As dynamic epigenetic alterations are coupled with transcriptional changes, we next examined chromatin conformation states. We found that the pericentromeric heterochromatin region (PHC), which normally showed intense DAPI staining, dramatically differed between Oct4-OE (high) and control. At the 2-cell stage, PHC of normal embryos forms a ring-like structure (Puschendorf *et al*. 2008), exhibited by most control embryos in this study by 30 h after insemination (Fig. 6A). However, in Oct4-OE, a dot pattern (chromocenter-like structure) rather than a ring-like formation developed (Fig. 6A). At the 2-cell stage, PHC is specified by H3K9me3 and Ring1b (Puschendorf *et al*. 2008); this modification pattern was also observed for Oct4-OE (Fig. 6B and C). The major satellite DNA was enriched at PHC in both groups (Fig. 6D). We also examined DNA methylation/hydroxylation states by IF, but observed no differences between Oct4-OE (high) and controls (Supplementary Fig. 11), suggesting that global DNA methylation might not be affected. Thus, high levels of exogenous OCT4 expression induced marked alteration of chromatin conformation without influencing its major components.

### Exogenous Oct4 expression accelerates cell cycle progression

**Figure 7:**
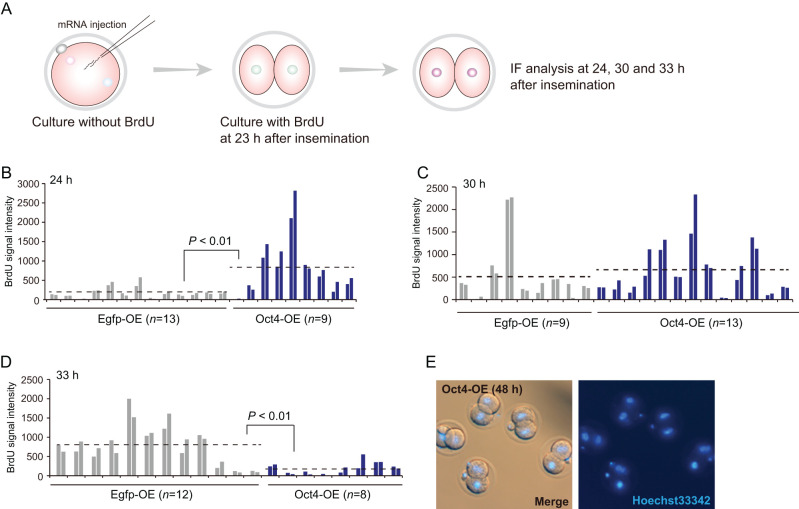
BrdU incorporation assay in Oct4 overexpressing 2-cell embryos. (A) The experimental scheme of the BrdU incorporation assay. (B–D) Signal quantification by IF analysis using anti-BrdU at 24 (B), 30 (C) and 33 h (D) after insemination. ‘n’ indicates the number of embryos. Broken lines indicate average signal levels and P-values were based on the Student’s *t*-test. (E) Hoechst33342 staining analysis of 2-cell-arrested Oct4-OEs. Arrested embryos were analyzed at 48 h after insemination. The nuclei show formation of a nuclear membrane, indicating that the cell cycle was in interphase.

Because RNA-seq analysis also identified cell-cycle-related genes (Fig. 4A), we analyzed the cell cycle states using bromodeoxyuridine (BrdU) incorporation followed by IF (Fig. 7A). At 24 h, most control embryos showed a weak BrdU signal (Fig. 7B). However, the majority of Oct4-OE (high) embryos showed extensive incorporation (Fig. 7B). At 30 h, no marked differences were found between groups (Fig. 7C). At 33 h, DNA synthesis occurred in most control embryos, whereas BrdU incorporation was not observed in Oct4-OE (high) (Fig. 7D). Given that fertilization timing was the same in Oct4-OE and the control group, these results indicate that ectopic OCT4 expression accelerated cell cycle progression.

To examine whether Oct4-OE (high) enter metaphase, we stained the embryos with Hoechst33342 at 48 h after insemination. As shown Fig. 7E, nuclear envelop breakdown did not occur, and chromatin condensation was not observed (Fig. 7E). Since BrdU was incorporated in Oct4-OE (Fig. 7B), Oct4-OE (high) were probably arrested at the G2 stage. Thus, OCT4 overexpression disrupted proper developmental programming.

## Discussion

**Figure 8:**
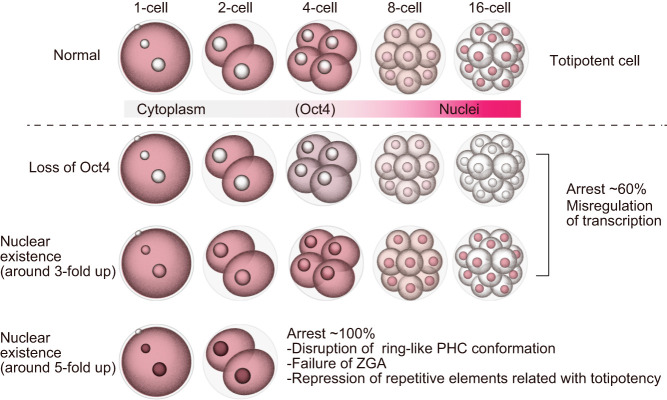
Model for the establishment of totipotency by OCT4 expression states. The nuclear presence of OCT4 and its level govern developmental competency.

In this study, we revealed that OCT4 protein localization was markedly altered during the epigenetic reprogramming phases after fertilization. Furthermore, exogenous *Oct4* expression impeded development in a dose-dependent manner. In murine ES cells, *Oct4* defines pluripotency in a dose-dependent manner (Niwa *et al*. 2000). This study demonstrated that this principal is also true for cells *in vivo*. A high dose of Oct4-OE resulted in dysregulation of ZGA-related genes and of certain types of repetitive sequences at the 2-cell stage (Figs 4 and 5), suggesting that these were the major causes of developmental arrest, whereas a low dose of Oct4-OE and Oct4 KD embryos led to the developmental failure at the late preimplantation phase (Fig. 3). At the late preimplantation phases, numerous pluripotency-, epigenome-, and differentiation-related genes are activated (Hamatani *et al*. 2004). Conversely, the dysregulation of DNA methylation-related factors and *Eomes*, associated with trophectoderm development (Russ *et al*. 2000), were observed in Oct4 KD (Supplementary Fig. 7B). Thus, we believe that these integral abnormalities of DNA methylation and proper lineage commitment might result in developmental arrest. However, we observed no marked changes in the expression of major transcription and epigenomic factors in Oct4-OE (low) (Supplementary Fig. 7B), suggesting that the reasons underlying developmental arrest differ and that the transcriptome analysis might help identify dysregulated genes in Oct4-OE (low). These results clearly indicated the importance of maintaining proper OCT4 expression levels for proper embryonic development. Moreover, it has been reported that ectopic expression of OCT4 *in vivo* causes dysplasia in epithelial tissues (Hochedlinger *et al*. 2005). Thus, the spatiotemporal regulation and fine-tuning of OCT4 protein is essential for cellular reprogramming and integrity (Fig. 8). Our findings also suggested that the silencing of *Oct4* function caused by its absence in the nucleus during early preimplantation phases might be essential for totipotency acquisition in early preimplantation development.

In this study, we found that exogenous expression of OCT4 resulted in marked changes in heterochromatin conformation (Fig. 6A), indicating that Oct4 potentially regulates chromatin states. Consistent with this notion, OCT4 has been shown to play an important role in chromatin opening at the 8-cell stage (Lu *et al*. 2016). Whether OCT4 directly mediated chromatin alteration remains unknown. However, recently, Nanog was shown to regulate heterochromatin organization by directly binding to pericentromeric regions in ES cells (Novo *et al*. 2016). Thus, OCT4 might control chromatin states via direct binding at many promoter regions. Consistent with this notion, we observed changes in the expression of many OCT4-binding genes (Fig. 4C).

We also found that the dynamic alteration of chromatin configuration in Oct4-OE (high) at the 2-cell stage was accompanied by the loss of the ring-like structure and resulted in a dot-like structure at pericentromeric regions (Fig. 6A). The dot-like structure at constitutive heterochromatin is a feature of embryos at the 4-cell stage onward (Puschendorf *et al*. 2008). Moreover, given that the S phase of the cell cycle in Oct4-OE proceeded faster than that in the control at 30 h after insemination (Fig. 7B–D), at which point a clear dot-like structure in Oct4-OE was observed (Fig. 6A), cell cycle acceleration by Oct4-OE (high) would result in chromatin alteration.

One of the remaining questions is what mechanisms are involved in OCT4 protein localization? The Oct4-C10 antibody was frequently reported (Wu *et al*. 2013, Bedzhov & Zernicka-Goetz 2014, Chen *et al*. 2015), and we also confirmed the specificity by SDS-WB using Oct4-KD and Oct4-OE embryos (Supplementary Fig. 12). In oocytes, the splice variants were not identified by RNA-seq analysis (Supplementary Fig. 4). Using RNA-seq data in 2-cell embryos, we confirmed no splice variants created alternative form of OCT4 protein in the 2-cell embryos (Supplementary Fig. 13). Therefore, the possibility that OCT4B was predominantly expressed in early preimplantation phases like humans was denied.

Another possibility is that OCT4 conformation might differ between early and late preimplantation phases. To test this, we conducted native-PAGE followed by WB analysis using embryos showing OCT4 negative staining by IF. However, OCT4 protein was detected in oocytes and 2-cell embryos (Supplementary Fig. 14A), suggesting that there are other causes for its differential localization, e.g. differences in post-translational modification such as phosphorylation of OCT4, which was shown to affect protein localization (Lin *et al*. 2012). Recently, the phosphorylation of nuclear localization signal has been important for shuttling between the nucleus and cytoplasm in RNF12/RLIM, essential for X chromosome inactivation (Jiao *et al*. 2013). To test the possibility, we conducted Phos-tag SDS-WB analysis using preimplantation embryos and the nuclei and cytoplasm of Oct4-OE. The results showed no clear difference in the band shift pattern among the preimplantation embryos (Supplementary Fig. 14B). Thus, other post-translational modifications or nuclear-cytoplasmic shuttling proteins might regulate Oct4 protein localization in preimplantation embryos.

The time at which OCT4 cytoplasmic localization begins also remains unknown. In this study, we demonstrated that OCT4 nuclear localization caused a dynamic change in chromatin conformation. Interestingly, this dynamic chromatin remodeling was observed in germ line reprogramming phases (Saitou *et al*. 2012), and *Oct4* has been used as a marker for primordial germ cells (Sugimoto & Abe 2007, Kobayashi *et al*. 2013). However, these studies were based on *Oct4* transgenic mice, which have exogenous *Oct4* promoter sequences with a GFP marker (Yoshimizu *et al*. 1999). Therefore, whether internal OCT4 protein localizes to the nucleus in primordial germ cells is unknown. A previous study using mice with *Oct4* knockout in primordial germ cells found that OCT4 deletion around embryonic day (E) 10.5 caused germ cell loss (Kehler *et al*. 2004), suggesting that OCT4 localizes to the nucleus until E10.5. Another IF-based study showed that OCT4 protein expression localized to the nucleus of germ cells at E12.5, although the antibody used in the study was not Oct4-C10. Nevertheless, considering that the antibody identified the nuclear OCT4 protein (Ma *et al*. 2014), it can be concluded that OCT4 protein localizes to the nucleus of germ cells until E12.5. Therefore, scrutinizing the expression states by IF during germ cell development will be informative and will provide new aspects of *Oct4*-mediated reprogramming machinery.

## Supplementary data

This is linked to the online version of the paper at http://dx.doi.org/10.1530/REP-16-0277.

## Declaration of interest

The authors declare that there is no conflict of interest that could be perceived as prejudicing the impartiality of the research reported.

## Funding

This work was supported by grants from the Ministry of Education, Culture, Sports, Science and Technology (MEXT) of Japan; a grant from the Ministry of Health, Labor and Welfare (MHLW) to H A and A U; a Grant-in-Aid for Scientific Research (21390456); a grant from JST-CREST to H A; and a JSPS KAKENHI Grant-in-Aid for Young Scientists (B) to A F (26861350).
